# Field clinical study evaluating the efficacy and safety of an oral formulation containing milbemycin oxime/praziquantel (Milbemax®, Novartis Animal Health) in the chemoprevention of the zoonotic canine infection by *Dirofilaria repens*

**DOI:** 10.1186/1756-3305-7-347

**Published:** 2014-07-29

**Authors:** Angela Di Cesare, Gabriele Braun, Emanuela Di Giulio, Barbara Paoletti, Vincenzo Aquilino, Roberto Bartolini, Francesco La Torre, Silvana Meloni, Jason Drake, Federico Pandolfi, Stefania Avolio, Donato Traversa

**Affiliations:** Faculty of Veterinary Medicine, University of Teramo, Piazza A. Moro, 45, 64100 Teramo, Italy; Klifovet AG, Munich, Geyerspergerstrasse 27, 80689 Munich, Germany; Veterinary Practice James Herriot, Via Nazionale 2/g, 64026 Roseto degli Abruzzi Teramo, Italy; Novartis Animal Health S.p.A., Largo Umberto Boccioni 1, Origgio, 21040 Varese, Italy; Novartis Animal Health, 3200 Northline Ave., Suite 300, Greensboro, NC 27408 USA; Canile Federico I°, Via San Giovanni 12/A, 60010 Ostra Vetere Ancona, Italy

**Keywords:** *Dirofilaria repens*, Milbemycin oxime, Prevention, Dog, Italy

## Abstract

**Background:**

*Dirofilaria repens* is the causative agent of subcutaneous dirofilariosis of dogs, other animals and humans. This nematode is transmitted by mosquitoes of *Aedes*, *Anopheles* and *Culex* genera. In dogs, the parasite may cause subclinical infection or cutaneous signs. Recently, *D. repens* has emerged and spread in different geographical areas, with an increase of cases in dogs and humans. Chemoprevention in dogs in endemic areas is the most reliable approach for controlling this infection. This paper describes a randomized, blocked and multicentric clinical field study investigating the efficacy of an oral, chewable formulation containing milbemycin oxime/praziquantel (Milbemax®, Novartis Animal Health) in the chemoprevention of subcutaneous dirofilariosis in dogs.

**Methods:**

This study was conducted in endemic areas of Italy. A total of 249 dogs, at two sites, negative for *D. repens,* were allocated into two groups (i.e. Treated -T1 *vs* Untreated-T2) with a ratio of 1:1, and subjected to clinical visits and blood sampling once monthly until the end of the study. All blood samples were microscopically and genetically examined. Animals belonging to T1 group received a minimum target dose of 0.5 mg/kg bodyweight of milbemycin oxime and 5 mg/kg of praziquantel in commercial tablets (Milbemax®) according body weight once every 4 weeks. Animals of group T2 were not treated with Milbemax® but received, when necessary, specific parasiticide treatments. The study duration was 336 ± 2 days for each dog.

**Results:**

A total of 219 dogs completed the study (i.e. 111 in T1 and 108 in T2), while 30 dogs (i.e. 13 in T1, 17 in T2) were withdrawn for a variety of reasons unrelated to administration of Milbemax®. The percentages of animals not showing microfilariae of *D. repens* were 100% (111 animals) in T1 and 94.7% (108 animals out of 114) in group T2. Milbemax® was shown to be safe in treated dogs.

**Conclusions:**

The results of this study confirm that the monthly use of Milbemax® in dogs is effective and safe for the prevention of subcutaneous dirofilariosis in endemic areas.

## Background

The nematode *Dirofilaria repens* (Nematoda, Onchocerciidae), the causative agent of subcutaneous dirofilariosis in dogs and other animals, is transmitted by bites of mosquitoes belonging to different genera, e.g. *Aedes*, *Anopheles* and *Culex* [1, 2]. The adult stages live in the subcutaneous tissues of the definitive host and, after mating, the females release the first stage larvae (microfilariae -mff) into the bloodstream of the infected animal [3].

In dogs, the infection may be asymptomatic although cutaneous signs of varying severity, such as dermatitis, (sub)-cutaneous nodules and lesions, itching and various allergic reactions could be caused by both adult stages and/or circulating mff [3–6]. Importantly, this nematode has a zoonotic potential and the human infection usually presents with subcutaneous nodules, pruriginous urticarioid patches, transient swellings and eosinophilia, although photophobia, conjunctival irritation and nodules or cysts in the eye or in the periocular tissues have also been reported [7, 8].

Canine subcutaneous dirofilariosis is distributed throughout the entire European territory, with different prevalence rates according to the geographical region [2, 9, 10]. While the occurrence of *D. repens* in southern Europe is well known [2, 11, 12], in the last few years the nematode has spread in previously free regions of eastern and central regions of the Old Continent, e.g. Austria [13, 14], Czech Republic [15], Germany [16–18], Hungary [19], Slovak Republic [20] and Ukraine [21]. Importantly, the number of human cases is increasing in several countries, e.g. Italy, Slovak Republic, Hungary and Poland [3, 7, 8, 22–25].

The spread of *D. repens* has likely been nurtured by a variety of factors, e.g. climatic changes, increased transport of goods across the world, reduction of movement restrictions of animals, movements of pets from endemic to free regions (e.g. adoption of infected animals and travels of companion animals with their owners), changes in the distribution of arthropods and pathogens they may transmit, as demonstrated by the case of the Asian tiger mosquito *Aedes albopictus* [1, 2, 26–28]. Treatment and control of *D. repens* is pivotal to reduce the transmission in dogs and to minimize the risk of infection in humans. The elimination of adult stages and circulating mff in infected dogs (e.g. *via* the administration of macrolactones) may interrupt the transmission of *D. repens* to the vectors [12, 19, 29, 30]. However, the most reliable approach to control canine dirofilarioses is the chemoprevention in healthy dogs living in endemic areas. While several molecules are licensed to prevent the canine heartworm *Dirofilaria immitis* [2, 3, 31], little information is available regarding the prevention of subcutaneous infections by *D. repens*. At this time, oral ivermectin and injectable and spot-on moxidectin are licensed for the prevention of subcutaneous dirofilariosis in dogs [3, 30]. Oral products containing milbemycin oxime are effective for the prevention of cardiopulmonary filariosis in dogs [3, 32, 33] but the usefulness of this macrocyclic lactone in the chemoprophylaxis of *D. repens* is practically unknown. Thus, the present clinical field study evaluated the efficacy and safety of a monthly administration of an oral, chewable formulation containing milbemycin oxime in the chemoprevention of *D. repens* infection in dogs living in endemic areas.

## Methods

### Authorizations and ethical statements

The study was carried out according to the European and national regulatory requirements and in compliance with the principles as on the follows:

- Directive 2001/82/EC as amended by 2004/28/EC and 2009/9/EC;
- VICH GL9 (GCP 6.June 2000) (CVMP/VICH/595/98 – FINAL): Guideline on good clinical practice for the conduct of clinical trials for veterinary medicinal products;
- EMEA/CVMP/816/00-Final: Guideline on statistical principles for veterinary clinical trials,
- VICH GL7 “Guideline on efficacy of anthelmintics, general requirements” (Step 7, CVMP/VICH/832/99-corr);
- VICH GL19 “Guideline on efficacy of anthelmintics, specific recommendations for canines” (Step 7, CVMP/VICH/835/99-Final);
- World Association for the Advancement of Veterinary Parasitology (W.A.A.V.P.) guidelines for evaluating the efficacy of anthelmintics for dogs and cats (Accepted 31 August 1993);
- The rules governing medicinal products in the European Union, Volume VII: Guidelines for the testing of veterinary medicinal products: Anthelmintics for cats and dogs: Specific requirements, (EudraLex guideline 7AE13a);

The study design and procedures were approved and authorized by the Italian Ministry of Health (date and authorization number 31.05.2012, 0010252-P). Competent local veterinary services (Azienda Sanitaria Locale -A.S.L.) were notified (04APR12 and 20.12.2014- 0148890, respectively) and no objections were raised.

No dogs included in the trial received any parasiticide with repellent activity, thus they were treated monthly with a spot-on combination of metaflumizone and amitraz (Promeris®, Zoetis) for the control of major ectoparasites and received a vaccine against *Leishmania infantum* (CaniLeish®, Virbac) according to manufacturer’s instructions.

### Sites, animals and study design

The trial was a blinded, randomized, blocked and clinical field study evaluating the preventive efficacy and safety of an oral, chewable formulation containing 0.5 mg/kg milbemycin oxime and 5 mg/kg praziquantel (Milbemax®, Novartis Animal Health) against subcutaneous dirofilariosis in dogs living in an endemic area of Italy, compared to an untreated control group.

The study was carried out from April 2012 to July 2013 and in two sites of the Abruzzo region, central Italy: a shelter located in Castelbasso municipality (Site A), and a private practice located in Roseto degli Abruzzi municipality (Site B). In total, 233 dogs at Site A and 16 dogs at Site B, that were both microscopically and molecularly (sections 2.2 and 2.3) negative for *D. repens* were enrolled. These dogs were evaluated for inclusion/exclusion criteria (section 2.2.1) and then definitively enrolled in the study on Day 0.

Dogs were allocated into two groups (i.e. Treated -T1 *vs* Untreated-T2) with a ratio of 1:1, and subjected to clinical observations and blood sampling once monthly until the end of the study.

Animals belonging to T1 group received a minimum target dose of 0.5 mg/kg bodyweight of milbemycin oxime and 5 mg/kg of praziquantel. The product was administered using commercial tablets in the appropriate size and amount of tablets for the animal weight band (based on the actual body weight) once every 4 weeks. The total duration of the study was 336 ± 2 days for each dog.

#### Screening: inclusion and exclusion criteria

Dogs were enrolled when fulfilling the following inclusion criteria:

- weighing ≥ 1.0 kg - 75.0 kg;
- living and/or frequently walking in an area with a prevalence of mosquitoes known to transmit *D. repens*;
- negative for microfilariae of *D. repens* and *D. immitis* upon modified Knott’s test and PCR at a screening performed one month prior to enrolment;
- treated with commercial Milbemax® no sooner than 4 weeks prior to enrolment in the study;
- clinically free of signs of infections with *D. repens*;
- suitable for the study when examined physically on Day 0;
- written consent signed by the owner or by an authorized representative.

Animals which did not fulfil the inclusion criteria or which showed one of the following exclusion criteria were excluded from the study:

- unstable health condition needing veterinary treatments prior to enrolment;
- clinical signs of infections with *D. repens* and/or *D. immitis*;
- treated with a product that has efficacy on *D. repens* (e.g. all macrocyclic lactones) less than 4 weeks prior to study start;
- dogs which received a repellent treatment within the last 14 days prior to enrolment;
- animals with any history of apparent reactions to Milbemax® or any of its compounds.
- animals with pre-existing medical and/or surgical conditions.

### Sample collection and laboratory examinations

#### Samples

Blood samples were collected from each dog into EDTA tubes (2.5 ml) at days 28 ± 2, 56 ± 2, 84 ± 2, 168 ± 2, 196 ± 2, 224 ± 2, 252 ± 2,280 ± 2, 308 ± 2 and 336 ± 2.

Samples were identified individually by unique identification numbers and stored at +4°C until direct transport within one day to the laboratory. According to the circadian rhythm of *D. repens* microfilariaemia [34] the collection was performed, in the morning before 10:00 o’clock or in the evening after 18:00 hours.

#### Microscopic methodologies

All blood samples were subjected to a qualitative modified Knott’s technique as previously described in the literature [35, 36]. Microfilariae were identified on the basis of differential morphometric (i.e. length and width) and morphological (i.e. head and tail) characteristics [36, 37]. For each positive sample the level of microfilariaemia was calculated using a slightly modified protocol described in the literature [38]. Briefly, one ml of blood was mixed with 9 ml of distilled water in a Falcon tube. The tube was thoroughly homogenized and an inverted McMaster Chamber was filled with a pipette. After two minutes the chamber was examined under a light microscope at 10X magnification and the mff were identified and counted. The number of mff counted in 0.30 ml of the McMaster chamber was multiplied × 30 (i.e. sensitivity for a 1:9 dilution) in order to obtain the number of mff in 1 ml of blood.

#### PCRs

The tubes containing the blood samples were left at room temperature for at least 20 minutes to allow the sedimentation of the mff, if present. An aliquot of ~150 μl of blood collected from the bottom of each tube was subjected to the DNA extraction using the commercial kit NucleosSpin® Blood (Macherey-Nagel, Duren, Germany). All DNA extracts were undertaken to semi-nested PCR protocols specific for mitochondrial gene encoding for subunit b of the cytochrome oxidase 1 (cox1) of canine filariae as previously described [31, 39]. In the first round, the universal primer set for spirurid nematodes COIintF (5′-TGATTGGTGGTTTTGGTAA-3′) and COIintR (5′-ATAAGTACGAGTATCAATATC-3′) were used. In the second round the specific reverse primers Dimm-cox1 R (5′-GCACTGACAATACCAAT-3′) for *D. immitis*, Drep-cox1 R (5′-TCAAACAGAAGTACCTAAA-3′) for *D. repens* and Drec-cox1 R (5′-CTGTGATGATTGGTTCT-3′) for *A. reconditum* were used, together with the COIintF forward primer. To confirm the identity of the species-specific PCRs, all amplicons visible on an agarose gel were purified over minicolumns (Ultrafree-DA, Millipore, Milan, Italy) and sequenced using a Taq DyeDeoxyTerminator cycle sequencing kit in an ABI-PRISM model 377 sequencer (Perkin Elmer, Warrington, UK). Accuracy was achieved by two-directional sequencing, and all electropherograms were manually checked and edited as necessary. Sequences were compared *in silico* with sequences of filarial mtDNA available in the GenBank™ using the Nucleotide-Nucleotide “Basic Local Alignment Search Tool” [40]. Once one microfilaria of *D. repens* had been detected by Knott’s test or in the case of positive result at PCR, the sample was regarded as positive.

### Physical examinations

For safety evaluation, during the study period, from Day of first treatment administration to study completion, all dogs treated with Milbemax® and all untreated animals were clinically observed within the scheduled study procedures. Additionally, owners were requested to inform the investigator whenever they observed any abnormalities in their animal(s). Any abnormality observed after the first treatment related or not to Milbemax® was recorded as an adverse event (AE). Any adverse event, which resulted in dead or in life-threatening or persistent or significant disability/incapacity was recorded as serious adverse event (SAE). Any AE suspected to be a drug reaction was recorded as suspected adverse drug reaction (SADR).

### Statistical analysis and efficacy evaluation

Data from all study animals were entered directly into an electronic data capture system (StudyBase®) purposed for the present work. At the end of the study all verified data were subjected to a statistical analysis.

All animals receiving at least one dose of Milbemax® and all control dogs were included in the safety analysis. In particular, all dogs with at least one post-baseline laboratory result for *D. repens* mff, and with a negative result for mff until day 168 ± 2 (i.e. 6 months after the first possible infection in 2012) formed the Intention-to-Treat (ITT) population. Animals with a positive result for *D. repens* before day 168 ± 2 were withdrawn from the study as they turned out as having been positive for *D. repens* on Day 0, and data of these animals were excluded from the ITT population.

Animals with premature discontinuation of the study for a reason other than positive result for *D. repens* were also excluded from the ITT population. The ITT population was used for the analysis of efficacy.

The primary and secondary efficacy criteria which evaluated the superiority of Milbemax® compared to the untreated control group were: i) percentage of animals not showing mff of *D. repens* and ii) mean mff count as assessed on the day of study completion compared between treatment groups.

Fisher’s Exact tests and related confidence intervals were performed with SAS® univariate procedures in order to calculate summary statistics and confidence intervals for means (SAS 9.2 Help and Documentation, Cary, NC: SAS Institute Inc., Copyright 2010). All variables measured in the study were described descriptively, and stratified by treatment group. For continuous variables, the following descriptive statistics were calculated: mean, standard deviation, sample size, median, quartiles, minimum and maximum. For categorical or binary variables, absolute and relative frequencies were analyzed. The first efficacy criterion was statistically evaluated using the Fisher’s exact test with a 95% confidence interval, while for the second efficacy criterion dog groups were compared using a two-sample t-test, if the normality assumption was satisfied for the log-transformed data. Otherwise, the non-parametric Mann–Whitney U test was used. Arithmetic means were analysed with 95% confidence limits on a base of log-transformed data and for original counts. A 5% level of significance (two-sided, P < 0.05) was used to assess statistical differences. The safety criteria percentage of adverse events (AE) per treatment group, percentage of serious adverse events (SAE) per treatment group and percentage of suspected adverse drug reactions (SADR) per treatment group were analysed per treatment group comparison using Fisher’s exact test.

## Results and discussion

**Figure 1:**
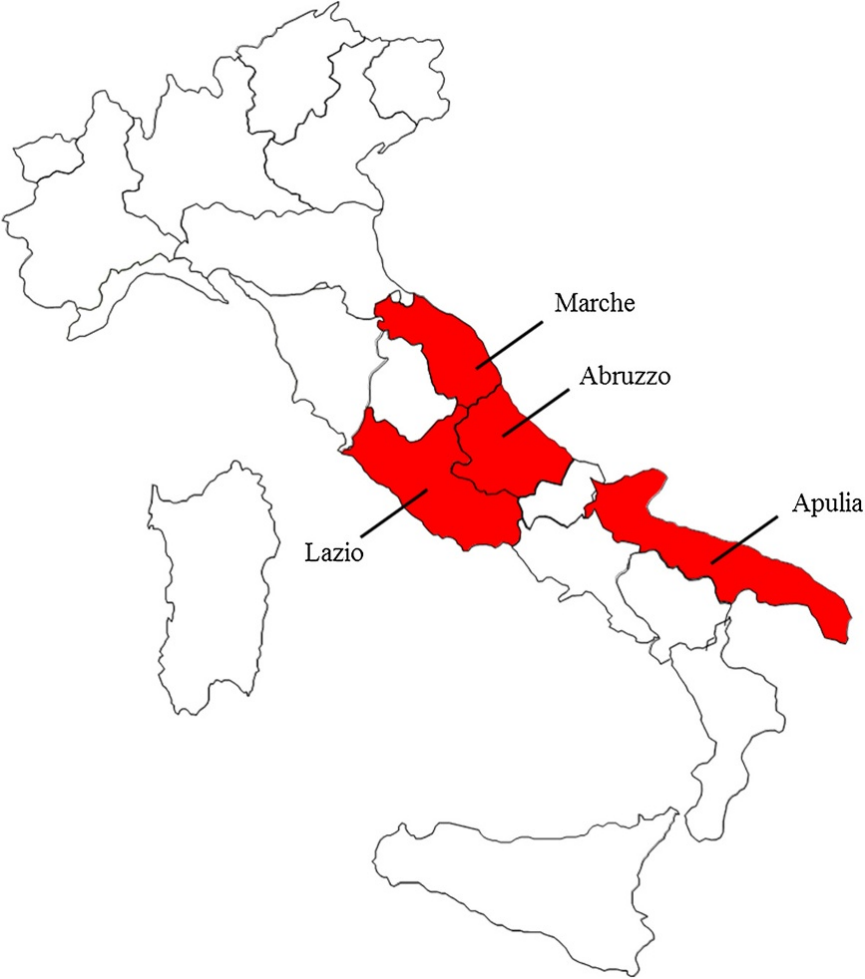
**Map of Italy.** Regions included in the study are indicated.

**Figure 2:**
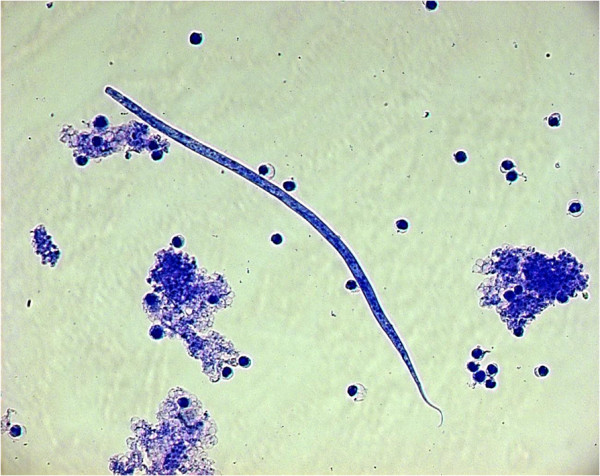
**Knott’s modified method: microfilaria of** ***Dirofilaria repens*** **.**

Overall, 116 and 117 dogs were enrolled in T1 and T2 at Site A, while 8 dogs were recruited in each treatment group at Site B. At site A, 10 dogs continued the study after adoption by private owners in the region of Abruzzo, and 34 animals continued the study after a transfer from site A to Marche region, northward from Abruzzo region. At site B, 2 privately owned dogs moved with their owners, one to the region Lazio and one to the region Apulia, and continued the study (Figure 1). The study was completed by 219 dogs (i.e. 111 in T1 and 108 in T2), while 30 dogs (i.e. 13 in T1, 17 in T2) were withdrawn either because they scored positive for *D. repens* (only for T2 group), i.e. 6 dogs, 4 at both microscopy (Figure 2) and PCR examinations and 2 only at the PCRs, or for other reasons (i.e. 24 dogs). The discrepancy in the microscopic and PCR results for T2 dogs which scored positive for *D. repens* during the study could be explained by a number of circulating mff lower than the sensitivity threshold of the Knott’s test.

None of the dogs belonging to T1 and T2 groups showed clinical signs related to the infection, and no mixed infections with other dog filariae were recorded in positive animals of the T2 group.

From the original population of 249 dogs, 244 were the animals with post-baseline laboratory results for microfilariae of *D. repens* and 19 of them were excluded from the ITT population.

Based on the ITT population, the percentages of animals testing negative for mff of *D. repens* (i.e. first criterion of efficacy) were 100% (111 animals) in T1 and 94.7% (108 animals out of 114) in T2. This difference was significant (p = 0.0293). With regard to the second criterion of efficacy, mff counts were assessed for the four dogs scored positive for the nematode at the Knott’s test, all in group T2. The arithmetic mean mff count was 18.0.

Although adverse events were observed in 16 (10 in T1 and 6 in T2) dogs during the study (e.g. gastric torsion, gastric and splenic torsion, bite wounds, tumours, natural death, dermatitis, giardiosis), none were regarded as related to the study medication. The percentage of animals with at least one adverse event and the percentage of animals with serious adverse events were comparable in both groups, (p = 0.3151 and p = 0.7688, respectively). This study reports the results of the first field investigation on the efficacy and safety of an oral, chewable formulation containing milbemycin oxime/praziquantel (Milbemax®, Novartis Animal Health) in the chemoprevention of the zoonotic canine infection by *D. repens*. The percentage of animals negative to *D. repens* demonstrated the superiority of Milbemax® in the prevention of subcutaneous dirofilariosis, compared to the untreated control group (p = 0.0293), in an endemic area. In fact, all T1-dogs (i.e. dogs which received the monthly anthelmintic formulation) remained negative for the presence of *D. repens* mff through the end of the study while 6 belonging to the untreated group T2 became infected during the study. This infection rate (i.e. 5.1%) is similar to that (i.e. 5.6%) described in a previous study carried out in the same area [41]. Importantly, all dogs that became microfilariemic received a rescue treatment, which showed a 100% efficacy in treating the infection (data not shown).

Different factors have recently promoted the spreading of *D. repens* and, indeed, the nematode is now emerging in several previously free areas [1, 26–28]. Chemoprevention by the administration of macrocyclic lactones in healthy dogs living in endemic areas is the most reliable approach to control canine filarioses and to minimize its zoonotic impact. In fact, macrocyclic lactones have several anti-nematode applications in small companion animal medicine and, importantly, they can be used as chemopreventatives against canine heartworm infection for their retroactive activity in killing third- and fourth-stage larvae. Specifically, oral (e.g. ivermectin, milbemycin oxime), topical (selamectin, moxidectin) or injectable sustained release (i.e. moxidectin) products are licensed for heartworm prevention in dogs and/or cats. Few of these molecules have been evaluated for their efficacy in preventing *D. repens* infection. Monthly administrations of oral ivermectin and moxidectin [42–45] and topical selamectin [11] have been reported to be effective to prevent *D. repens* infections in dogs. A single microsphere sustained release injectable moxidectin treatment at the dose of 0.17 mg/kg was effective in assuring an at least 6-months lasting protection of dogs against *D. repens* [46]. Two recent trials have shown the preventive efficacy of monthly applications of 2.5% spot on moxidectin against *D. repens* in dogs, with a 100% efficacy of a single administration in experimental infections with infective larvae [30]. Moreover, the potential use of the same formulation in the simultaneous prevention of major dog filarioses (i.e. *D. immitis*, *D. repens*, and *Acanthocheilonema reconditum*) has been demonstrated preliminarily [47].

The present study provides evidence for the first time that oral milbemycin oxime is both effective and safe in the chemoprevention of *D. repens* infection in dogs living in an endemic area when administered once every 30 days. Importantly, the frequency of periodic oral dosing with milbemycin oxime to prevent *D. repens* is aimed not at maintaining steady levels of the compound in the blood or tissues of the host, but rather to obtain a retroactive efficacy on third and fourth-stage larvae in the tissue of dogs which received bites from infected mosquitoes. Moreover, Milbemax® at the same dosage may provide control of major canine intestinal nematodes [48], *Spirocerca lupi* [49], the zoonotic eyeworm *Thelazia callipaeda* [50, 51], *Angiostrongylus vasorum* and *Crenosoma vulpis* [52, 53].

This broad spectrum of activity, which assures a monthly prevention of filariae and continued control of intestinal infections and other parasites, could make an “all year round” worm control program desirable. Indeed, in the USA, there is the frequent willingness of many pet owners to administer lifelong monthly treatments of macrocyclic lactones to provide reliable control against intestinal nematodes and *D. immitis* at the same time [48, 54]. Although the year-round approach is not currently applied in many parts of Europe and not recommended by The European Scientific Council of Companion Animal Parasites (ESCCAP), this approach may considered to be advisable in particular epidemiological settings to assure treatment, prevention and/or control of major canine parasites, including mosquito-borne nematodes. This is particularly important if one bears in mind that *D. repens* is spreading into geographic areas previously free of this parasite and the number of clinical cases of *D. repens* in dogs and humans is also rising in areas where canine heartworm (*D. immitis*) is endemic [2, 3, 10, 24].

## Conclusions

The results of this study demonstrate that the oral, chewable tablet containing milbemycin oxime and praziquantel (Milbemax®) provides a new and effective way to control canine subcutaneous filariosis, i.e. a zoonotic parasitosis, which is spreading in several European countries. Specifically, the prophylactic activity of Milbemax® in preventing *D. repens* is due to the macrocyclic lactone contained in the formulation.

A regular monthly treatment with Milbemax® has the potential to interrupt the life cycle of *D. repens*, with important implications under epidemiological and clinical standpoints. In fact, minimizing the number of infected dogs acting as reservoirs of the parasite is central to avoid infective blood meals for the mosquitoes and to reduce the occurrence of *D. repens* in their vectors, in dogs and humans. From a practical standpoint, the prevention of *D. repens* infection is important in canine clinical practice, as adult parasites and/or circulating mff may induce different cutaneous signs in infected dogs and pose challenging issues for the diagnosis and treatment of these dog dermatopathies [3–6].
